# Factors affecting post-pubertal penile size in patients with hypospadias

**DOI:** 10.1007/s00345-016-1763-0

**Published:** 2016-01-20

**Authors:** Kimihiko Moriya, Michiko Nakamura, Yoko Nishimura, Takeya Kitta, Yukiko Kanno, Hiroki Chiba, Masafumi Kon, Nobuo Shinohara

**Affiliations:** Department of Renal and Genitourinary Surgery, Hokkaido University Graduate School of Medicine, North-15, West-7, Kita-ku, Sapporo, 060-0824 Japan

**Keywords:** Long term, Post-pubertal penile size, Hypospadias

## Abstract

**Objectives:**

To evaluate actual post-pubertal penile size and factors affecting it in hypospadias patients, we retrospectively reviewed medical charts.

**Patients and methods:**

Hypospadias patients whose external genitalia were categorized into Tanner stage 5, and whose stretched penile length was evaluated at 15 years old or older from April 2008 to April 2015, were enrolled in the present study. Stretched penile length was measured by a single examiner. Actual post-pubertal stretched penile length and factors affecting the post-pubertal stretched penile length were estimated. Statistical analysis was performed using Mann–Whitney *U* test and univariate and multivariate linear regression models for the determination of independent factors.

**Results:**

Thirty patients met the inclusion criteria. Median age at evaluation was 17.2 years. Thirteen and 17 had mild and severe hypospadias, respectively. Endocrinological abnormality was identified in 5. Multivariate analysis showed that the severity of hypospadias and endocrinological abnormality were significant factors affecting stretched penile length. Stretched penile length in 25 patients without endocrinological abnormality was significantly longer than that in those with endocrinological abnormality (*p* = 0.036). Among patients without endocrinological abnormality, stretched penile length in 13 with severe hypospadias was significantly shorter than that in 12 with mild hypospadias (*p* = 0.004).

**Conclusions:**

While the severity of hypospadias and endocrinological abnormality at post-pubertal evaluation were factors affecting post-pubertal penile size, stretched penile length in patients with severe hypospadias was shorter even in cases without endocrinological abnormality. These results suggest that severe hypospadias is not only a disorder of urethral development, but also a disorder of penile development.

## Introduction

Recent studies regarding the long-term outcome of hypospadias surgery have shown that smaller penile size was one of the main complaints in patients at puberty or later [1–5]. Despite the anxiety of patients regarding penile size as reported in questionnaires in previous studies, reports of actual post-pubertal penile size in hypospadias patients are limited. In most of the literature, the penile length of patients who had hypospadias surgery in childhood was compared depending on the severity of hypospadias [6, 7]. In those studies, it was revealed that the severity of hypospadias was a significant risk factor for smaller penile size.

Since hypospadias is a complex congenital anomaly, some maternal factors affecting the occurrence [8] or severity [9] of it were reported, which may have potential to affect the post-pubertal penile size. Surgical procedure [10] or physical growth [11] as well as congenital factors may also have some influence on the post-pubertal penile size. Accordingly, not only the severity of hypospadias but also various other factors could affect the post-pubertal penile size, including maternal factors such as low birth weight, surgical factors such as dorsal plication of the penis for correction of curvature, endocrinological factors such as abnormality of the pituitary–gonadal axis at puberty [12], or factors regarding physical growth. In the present study, to clarify the factors affecting post-pubertal penile length, we retrospectively reviewed medical charts in order to evaluate actual post-pubertal penile length and factors affecting post-pubertal penile length in hypospadias patients.

## Patients and methods

All patients with a history of hypospadias surgery at our institute or affiliated hospitals were recommended to be followed up for regular visit until post-pubertal period. Medical chart of patients who visited our clinic for regular follow-up after hypospadias surgery was retrospectively reviewed. Then, patients who were operated at our institute or affiliated hospitals between December 1986 and December 2002, who were categorized into Tanner stage 5, and whose stretched penile length (SPL) was evaluated at 15 years old or older from April 2008 to April 2015 were enrolled in the present study. Patients without information regarding the preoperative severity of hypospadias and/or the surgical procedure at initial surgery, especially for the correction of chordee deformity, were excluded from this study. If multiple evaluations of SPL were carried out in one patient who was categorized into Tanner stage 5 at 15 years old or older, the final evaluation was used for the data in the present study. SPL was measured as reported by Wessells et al. [13] by a single examiner (KM). The severity of hypospadias was divided into mild and severe, based on Koyanagi’s classification [14]. Briefly, when no or mild chordee deformity was identified after degloving, the cases were diagnosed as mild hypospadias. When transection of the urethral plate was required to correct moderate to severe chordee deformity which was confirmed after degloving, the cases were diagnosed as severe hypospadias. Endocrinological abnormality was defined as hypersecretion of luteinizing hormone and/or low serum testosterone at the evaluation of SPL. Factors affecting the post-pubertal SPL were estimated. Statistical analysis was performed using Mann–Whitney *U* test and univariate and multivariate linear regression models for the determination of independent factors. *p* < 0.05 was considered statistically significant.

## Results

Thirty patients met the inclusion criteria. Patient characteristics are shown on Table 1. Median birth weight was 2330 g (unknown in 3). Thirteen and 17 had mild and severe hypospadias, respectively. Surgical procedures for urethroplasty were onlay urethroplasty in 11 and tubularized incised plate urethroplasty in 2 for mild hypospadias, and Koyanagi procedure in 16 and tube island flap in 1 for severe hypospadias. Dorsal tunica albuginea plication was performed for the correction of chordee deformity in 1 with mild hypospadias and 4 with severe hypospadias. Unilateral and bilateral undescended testes were surgically managed in 4 and 1, respectively. The median number of surgery for hypospadias was 2. Median age at the evaluation of SPL was 17.2 years. Mean SPL was 111.1 mm among all patients. Endocrinological abnormality at the time of SPL measurement was identified in 5 patients (1 with mild hypospadias and 4 with severe hypospadias). Among them, hypogonadotropic hypogonadism, hypergonadotropic hypogonadism, and normogonadotropic hypogonadism were identified in 2, 1, and 2, respectively.

**Table 1:** Patients’ characteristics

		Range
Birth weight (g) (median ± SD)	2330 ± 770 (unknown: 3)	738–3638
Severity of hypospadias	Mild: 13, severe: 17	
Age at initial surgery (year) (median ± SD)	2.4 ± 0.9	1.4–6.3
Surgical procedure for urethroplasty	Onlay: 11, TIP^a^: 2, Koyanagi: 16, island flap (tube): 1	
Dorsal plication at surgery	5 (ventral lengthening: 0)	
History of undescended testis	5 (unilateral: 4, bilateral: 1)	
The number of surgery (median ± SD)	2 ± 2.2	1–12
Age at evaluation (year) (median ± SD)	17.2 ± 2.8	15.0–29.5
Height at evaluation (cm) (median ± SD)	163.9 ± 8.4 (unknown: 2)	149.0–178.5
Stretched penile length (mm) (mean ± SD)	111.1 ± 25.1	45–155
Endocrinological abnormality	5^b^	

^a^Tubularized incised plate urethroplasty

^b^Hypogonadotropic hypogonadism, hypergonadotropic hypogonadism, and normogonadotropic hypogonadism were identified in 2, 1, and 2, respectively

Univariate analysis showed that the severity of hypospadias and endocrinological abnormality were significant factors for post-pubertal SPL. On multivariate analysis, both the severity of hypospadias and endocrinological abnormality were also significant factors for post-pubertal SPL. Birth weight, age at initial surgery, history of dorsal tunica albuginea plication at surgery, history of undescended testis, the number of surgery and age and height at evaluation did not affect post-pubertal SPL (Table 2). SPL in 25 patients without endocrinological abnormality (mean 115.7 mm) was significantly longer than in those with endocrinological abnormality (88.0 mm) (*p* = 0.036) (Table 3). Among 25 patients without endocrinological abnormality, SPL in 13 with severe hypospadias (102.5 mm) was significantly shorter than that in 12 with mild hypospadias (129.9 mm) (*p* = 0.004) (Table 4).

**Table 2:** Univariate and multivariate linear regression analyses of factors affecting stretched penile length

	Univariate		Multivariate	
	Coefficient (95 % CI)	*p* value	Coefficient (95 % CI)	*p* value
Birth weight	−0.0001 (−0.013, 0.0133)	0.981		
Severe hypospadias	−15.484 (−23.010, −7.958)	<0.001	−13.910 (−21.185, −6.633)	<0.001
Age at initial surgery	−0.580 (−1.429, 0.269)	0.173		
Dorsal plication at surgery	−7.710 (−21.781, 6.361)	0.271		
History of undescended testis	−5.050 (−17.366, 7.266)	0.405		
The number of surgery	−0.688 (−5.136, 3.760)	0.754		
Age at evaluation	−2.788 (−6.081, 0.504)	0.094		
Height at evaluation	0.895 (−0.287, 2.078)	0.132		
Endocrinological abnormality	−13.840 (−25.457, −2.23)	0.021	−9.945 (−19.620, −0.271)	0.044

**Table 3:** Stretched penile length in patients with and without endocrinological abnormality

	Stretched penile length (mm) (mean ± SD)	*p* value
Patients without endocrinological abnormality (*n* = 25)	115.7 ± 23.7	0.036
Patients with endocrinological abnormality (*n* = 5)	88.0 ± 19.7

**Table 4:** Stretched penile length depending on severity of hypospadias

	Stretched penile length (mm) (mean ± SD)	*p* value
*All patients*		
Mild hypospadias (*n* = 13)	128.6 ± 19.6	<0.001
Severe hypospadias (*n* = 17)	97.6 ± 20.2	
*Patients without endocrinological abnormality*		
Mild hypospadias (*n* = 12)	129.9 ± 19.9	0.004
Severe hypospadias (*n* = 13)	102.5 ± 19.2	

## Discussion

Previous questionnaire-based studies demonstrated that smaller penile size was one of the main complaints in patients at puberty or later [1–5]. However, as is commonly known as “small penis anxiety” [15] or “small penis syndrome” [16], some men with no history of penile disease are distressed by the size of their penis, even when it is within the normal range. Men with a history of hypospadias surgery might be more sensitive regarding the appearance of their penis, which would have affected the outcome of the questionnaire-based studies. By measuring the actual penile size and evaluating the factors affecting penile size, we could shed light on new aspects of hypospadias in the long term from a different point of view.

Actual SPL was evaluated at our clinic by one examiner in the present study in order to avoid bias between examiners and the unreliability of SPL due to self-reporting. Only patients who were categorized into Tanner stage 5 at 15 years or older were included in the present study, based on the recommendation of Soydan et al. [17] that penile length be evaluated according to the pubertal stage because those within the same pubertal stage were similar. By limiting patients only to those in Tanner stage 5 and using one examiner to minimize the bias, actual post-pubertal penile size and factors affecting post-pubertal penile size could be evaluated quite precisely in the present study.

Our data demonstrated that the severity of hypospadias and endocrinological abnormality were significant factors affecting penile size. In cases with mild hypospadias without endocrinological abnormality, mean SPL was 129.9 mm, which was almost the same as that of adult men without penile abnormality (mean 132.4 mm), as demonstrated by Veale et al. [11]. In addition, this finding is consistent with a previous report by Ortqvist et al. [7]. This finding suggests that penile development is not or minimally impaired in cases with mild hypospadias unless the pituitary–gonadal axis is maintained. In contrast, SPL in cases with severe hypospadias was significantly shorter than that with mild hypospadias, even in cases without endocrinological abnormality. Although Fievet et al. [18] demonstrated that penile length measured in childhood was comparable in boys with and without hypospadias regardless of the degree of hypospadias, the outcomes of several studies in which SPL was evaluated after puberty were consistent with our data [6, 7]. This suggests that penile development is impaired along with urethral development in cases with severe hypospadias. Since the cause of hypospadias is still obscure in the majority of cases [19], the relationship between penile and urethral development in cases with hypospadias also remains unknown. Further studies are necessary to clarify the mechanism of impairment in penile development in patients with hypospadias.

Although post-pubertal penile size in hypospadias patients has been evaluated in several studies, endocrinological abnormality has not been pointed out as a factor affecting post-pubertal penile size in those patients because no study has reported endocrinological status at the time of evaluation of penile size at puberty [6, 7, 20]. Accordingly, this is the first report demonstrating endocrinological abnormality at puberty as a significant factor affecting post-pubertal penile size. As reported previously, post-pubertal endocrinological abnormality in the pituitary–gonadal axis was identified in some patients with hypospadias irrespective of its severity [12]. It is well known that prepubertal androgen administration contributes to penile growth in patients with hypospadias [21, 22]. In cases with hypogonadism without hypospadias, penile growth was observed by hormone replacement therapy even in the post-pubertal period [23, 24]. On the basis of these reports, hormone replacement therapy might be beneficial for penile growth depending on the status of endocrinological abnormality in cases with hypospadias. While endocrinological abnormality was identified in five cases in our study, no patients received hormone replacement therapy because they did not desire it.

Dorsal tunica albuginea plication was not a significant factor affecting penile length in the present study. While some worried about penile shortening associated with dorsal tunica albuginea plication [25–27], there has been no solid evidence that dorsal tunica albuginea plication performed in childhood significantly affected post-pubertal penile length or patients’ satisfaction regarding cosmesis. Our study included a limited number of patients who had severe hypospadias or who required dorsal tunica albuginea plication. In addition, no patients in whom a ventral lengthening procedure was indicated were enrolled in the present study. There might not have been sufficient statistical power to detect the impact of dorsal tunica albuginea plication. To clarify its impact or that of a ventral lengthening procedure in childhood for the correction of chordee deformity regarding actual penile size or patients’ satisfaction regarding cosmesis at puberty or later, a randomized study with a long-term follow-up should be performed.

There are several limitations to this study. First, while more than 200 patients were operated on in our institute or affiliated hospitals between December 1986 and December 2002, only 30 patients were included in the present study. Accordingly, our outcome might contain some referral bias. While we recommend regular visit until post-pubertal period to all patients after hypospadias surgery, many patients do not stay at the same place long after surgery and some patients or parents may refuse to visit hospital unless they have symptoms or are anxious. Long-term follow-up studies of hypospadias surgery are often difficult for all our efforts, especially which include physical examination and/or blood sampling like the present study. Second, the statistical power was limited because of the limited number of patients enrolled in the present study. Thus, other significant factors affecting post-pubertal penile size might have been overlooked. Third, SPL was evaluated by one examiner to avoid bias between examiners. However, since SPL was obtained between April 2008 and August 2015, inter-examination bias might not have been avoided.

In conclusion, while the severity of hypospadias and endocrinological abnormality at post-pubertal evaluation were factors affecting post-pubertal penile size, SPL in patients with severe hypospadias was shorter even in cases without endocrinological abnormality. This observation suggests that severe hypospadias would be not only a disorder of urethral development but also a disorder of penile development. In addition, the improvement in penile size might be achieved in some patients with endocrinological abnormality by correcting their endocrinological status.

## References

[1] Mureau MA, Slijper FM, Nijman RJ (1995). Psychosexual adjustment of children and adolescents after different types of hypospadias surgery: a norm-related study. J Urol.

[2] Aho MO, Tammela OK, Somppi EM (2000). Sexual and social life of men operated in childhood for hypospadias and phimosis. A comparative study. Eur Urol.

[3] Bubanj TB, Perovic SV, Milicevic RM (2004). Sexual behavior and sexual function of adults after hypospadias surgery: a comparative study. J Urol.

[4] Moriya K, Kakizaki H, Tanaka H (2006). Long-term cosmetic and sexual outcome of hypospadias surgery: norm related study in adolescence. J Urol.

[5] Ciancio F, Lo Russo G, Innocenti A (2015). Penile length is a very important factor for cosmesis, function and psychosexual development in patients affected by hypospadias: results from a long-term longitudinal cohort study. Int J Immunopathol Pharmacol.

[6] Rynja SP, Wouters GA, Van Schaijk M (2009). Long-term followup of hypospadias: functional and cosmetic results. J Urol.

[7] Ortqvist L, Fossum M, Andersson M (2015). Long-term followup of men born with hypospadias: urological and cosmetic results. J Urol.

[8] Brouwers MM, Feitz WF, Roelofs LA (2007). Risk factors for hypospadias. Eur J Pediatr.

[9] van Rooij IA, van der Zanden LF, Brouwers MM (2013). Risk factors for different phenotypes of hypospadias: results from a Dutch case-control study. BJU Int.

[10] Simonato A, Gregori A, Ambruosi C (2007). Congenital penile curvature: dermal grafting procedure to prevent penile shortening in adults. Eur Urol.

[11] Veale D, Miles S, Bramley S (2015). Am I normal? A systematic review and construction of nomograms for flaccid and erect penis length and circumference in up to 15 521 men. BJU Int.

[12] Moriya K, Mitsui T, Tanaka H (2010). Long-term outcome of pituitary–gonadal axis and gonadal growth in patients with hypospadias at puberty. J Urol.

[13] Wessells H, Lue TF, McAninch JW (1996). Penile length in the flaccid and erect states: guidelines for penile augmentation. J Urol.

[14] Koyanagi T, Nonomura K, Yamashita T (1994). One-stage repair of hypospadias: is there no simple method universally applicable to all types of hypospadias?. J Urol.

[15] Veale D, Miles S, Read J (2015). Phenomenology of men with body dysmorphic disorder concerning penis size compared to men anxious about their penis size and to men without concerns: a cohort study. Body Image.

[16] Wylie KR, Eardley I (2007). Penile size and the ‘small penis syndrome’. BJU Int.

[17] Soydan H, Akyol I, Ates F (2012). Cross-sectional analysis of penile length in males 13–15 years old according to pubertal development stages. J Urol.

[18] Fievet L, Harper L, Chirpaz E (2012). Penile length is comparable in boys with and without hypospadias. J Pediatr Urol.

[19] Kon M, Suzuki E, Dung VC (2015). Molecular basis of non-syndromic hypospadias: systematic mutation screening and genome-wide copy-number analysis of 62 patients. Hum Reprod.

[20] Aulagne MB, Harper L, de Napoli-Cocci S (2010). Long-term outcome of severe hypospadias. J Pediatr Urol.

[21] Sakakibara N, Nonomura K, Koyanagi T (1991). Use of testosterone ointment before hypospadias repair. Urol Int.

[22] Netto JM, Ferrarez CE, Schindler Leal AA (2013). Hormone therapy in hypospadias surgery: a systematic review. J Pediatr Urol.

[23] Kirk JM, Savage MO, Grant DB (1994). Gonadal function and response to human chorionic and menopausal gonadotrophin therapy in male patients with idiopathic hypogonadotrophic hypogonadism. Clin Endocrinol (Oxf).

[24] Kim SO, Ryu KH, Hwang IS (2011). Penile growth in response to human chorionic gonadotropin (HCG) treatment in patients with idiopathic hypogonadotrophic hypogonadism. Chonnam Med J.

[25] Chertin B, Koulikov D, Fridmans A (2004). Dorsal tunica albuginea plication to correct congenital and acquired penile curvature: a long-term follow-up. BJU Int.

[26] Kajbafzadeh AM, Arshadi H, Payabvash S (2007). Proximal hypospadias with severe chordee: single stage repair using corporeal tunica vaginalis free graft. J Urol.

[27] Castellan M, Gosalbez R, Devendra J (2011). Ventral corporal body grafting for correcting severe penile curvature associated with single or two-stage hypospadias repair. J Pediatr Urol.

